# 2-[2-Benzoyl-3,3-bis­(methyl­sulfan­yl)prop-2-enyl­idene]malononitrile

**DOI:** 10.1107/S1600536809024635

**Published:** 2009-07-01

**Authors:** Joseph Nirmala, N. V Unnikrishnan, E. R Anabha, C. Sudarsanakumar

**Affiliations:** a School of Pure and Applied Physics, Mahatma Gandhi University, Kottayam, Kerala, India; b School of Chemical Sciences, Mahatma Gandhi University, Kottayam, Kerala, India

## Abstract

The title compound, C_15_H_12_N_2_OS_2_, is an example of a push–pull butadiene in which the electron-releasing methyl­sulfanyl groups and electron-withdrawing nitrile groups on either end of the butadiene chain enhance the conjugation in the system. Short intra­molecular C—H⋯S inter­actions are observed. In the crystal structure, an O⋯C short contact of 2.917 (3) Å is observed.

## Related literature

The title compound was obtained during the synthesis of pyr­idene derivatives, see: Anabha & Asokan (2006 ▶). In push–pull butadienes, the C=C double bonds usually become more polarized due to π-electron delocalization (Dahne, 1978 ▶; Michalik *et al.*, 2002 ▶). For related structures, see: Dastidar *et al.* (1993 ▶); Freier *et al.* (1999 ▶); Homrighausen & Krause Bauer (2004 ▶). 
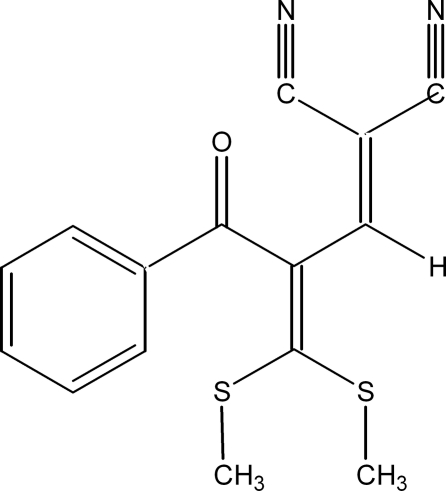


## Experimental

### 

#### Crystal data

C_15_H_12_N_2_OS_2_
*M* *_r_* = 300.39
Monoclinic, 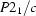

*a* = 5.6557 (2) Å
*b* = 8.5153 (3) Å
*c* = 31.4726 (11) Å
β = 90.106 (2)°
*V* = 1515.72 (9) Å^3^
*Z* = 4
Mo *K*α radiation
μ = 0.35 mm^−1^
*T* = 298 K
0.40 × 0.35 × 0.30 mm

#### Data collection

MacScience DIPLabo 32001 diffractometer
Absorption correction: none
9663 measured reflections
2816 independent reflections
2338 reflections with *I* > 2σ(*I*)
*R*_int_ = 0.024

#### Refinement

*R*[*F* ^2^ > 2σ(*F* ^2^)] = 0.039
*wR*(*F* ^2^) = 0.128
*S* = 1.13
2816 reflections
183 parameters
H-atom parameters constrained
Δρ_max_ = 0.26 e Å^−3^
Δρ_min_ = −0.25 e Å^−3^

### 

Data collection: *XPRESS* (MacScience, 2002 ▶); cell refinement: *SCALEPACK* (Otwinowski & Minor, 1997 ▶); data reduction: *SCALEPACK* and *DENZO* (Otwinowski & Minor, 1997 ▶); program(s) used to solve structure: *SHELXS97* (Sheldrick, 2008 ▶); program(s) used to refine structure: *SHELXL97* (Sheldrick, 2008 ▶); molecular graphics: *PLATON* (Spek, 2009 ▶) and *ORTEPII* (Johnson, 1976 ▶); software used to prepare material for publication: *SHELXL97* and *PLATON*.

## Supplementary Material

Crystal structure: contains datablocks global, I. DOI: 10.1107/S1600536809024635/ci2822sup1.cif

Structure factors: contains datablocks I. DOI: 10.1107/S1600536809024635/ci2822Isup2.hkl

Additional supplementary materials:  crystallographic information; 3D view; checkCIF report

## Figures and Tables

**Table 1:** Hydrogen-bond geometry (Å, °)

*D*—H⋯*A*	*D*—H	H⋯*A*	*D*⋯*A*	*D*—H⋯*A*
C10—H10*A*⋯S1	0.96	2.82	3.360 (3)	116
C12—H12⋯S1	0.93	2.66	3.040 (2)	105

**Fig. 1.**
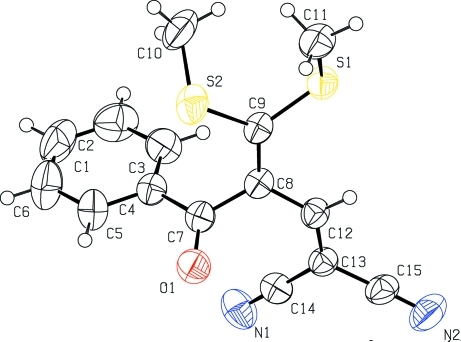
An ORTEPII (Johnson, 1976) view of the title compound, showing the atom-labeling scheme. Displacement ellipsoids are drawn at the 50% probability level.

## supplementary crystallographic information

### Comment

The title compound belongs to the class of push-pull butadiene as they have electron releasing methyl sulfanyl groups and electron withdrawing nitrile groups attached to the terminal carbon atoms of the butadiene moiety. The butadiene molecules are characterized by significant π-electron interactions between donor and acceptor groups and the diene double bond system. Usually C═C double bonds become more polarized due to π-electron delocalization (Dahne, 1978; Michalik *et al.*, 2002). They are important as pivots for the synthesis of heterocycles especially pyridine derivatives. The title compound was obtained during the synthesis of pyridene derivatives (Anabha *et al.*, 2006) and its crystal and molecular structure was determined to study the influence of aroyl group on the steriochemistry of the butadiene molecule.

A perspective view of the title molecule is shown in Fig.1. The two double bonds in the butadiene moiety are arranged in a *transoid* manner. The lengths of the C8—C9 [1.366 (3) Å] and C13—C12 [1.352 (3) Å] double bonds and C8—C12 [1.439 (3) Å] single bond indicate conjugation. The butadiene unit is almost planar as evidenced by the torsion angles C9—C8—C12—C13, C8—C12—C13—C14 and C12—C8—C9—S1 of -167.0 (2)°, 6.1 (4)° and 17.8 (3)°, respectively. The two methylsulfanyl groups (–SCH3) are oriented in such a way as to avoid the interaction between them as is evident from the torsion angles C10—S2—C9—C8 of -129.17 (19)° and C11—S1—C9—C8 of -148.69 (19)°. Crystal structures of other butadiene compounds reported also show similar geometric parameters (Dastidar *et al.*., 1993; Michalik *et al.*, 2002; Freier *et al.*,1999; Homrighausen *et al.*, 2004).

Weak intramolecular C—H···S interactions are observed (Table 1). In the crystal structure a O1···C14(1-x, y, z) short contact [2.917 (3) Å] is observed.

### Experimental

2-Benzoyl-2-[3,3-bis(methylsulfanyl)-2-propylidene]malononitrile was synthesized as follows: A mixture of malononitrile (500 mg, 7.5 mmol), ammonium acetate (1.5 g, 20 mmol) and acetic acid (5 ml) was heated to 343 K and then 2-benzoyl-3,3-bis(methylsulfanyl)acrylaldehyde (5 mmol) was added. The reaction mixture was stirred for 5 minutes at the same temperature, cooled to room temperature and then poured into ice-cold water. The solid separated was filtered, dissolved in chloroform, dried over anhydrous sodium sulfate and then the solvent was evaporated. The crude product obtained was recrystallized from hexane-ethyl acetate solvent mixture.

### Refinement

All H atoms were positioned geometrically and allowed to ride with parent atoms, with C-H distances of 0.93 or 0.96 A. Their isotropic displacement parameters were defined as *U*_iso_ = 1.5U_eq_(C) for the methyl H atoms and *U*_iso_ = 1.2U_eq_(C) for all other atoms.

### Crystal data

**Table:**

C_15_H_12_N_2_OS_2_	*F*(000) = 624
*M**_r_* = 300.39	*D*_x_ = 1.316 Mg m^−^^3^
Monoclinic, *P*2_1_/*c*	Mo *K*α radiation, λ = 0.71073 Å
Hall symbol: -P 2ybc	Cell parameters from 9663 reflections
*a* = 5.6557 (2) Å	θ = 1.3–25.5°
*b* = 8.5153 (3) Å	µ = 0.35 mm^−^^1^
*c* = 31.4726 (11) Å	*T* = 298 K
β = 90.106 (2)°	Block, pale yellow
*V* = 1515.72 (9) Å^3^	0.40 × 0.35 × 0.30 mm
*Z* = 4	

### Data collection

**Table:**

MacScience DIPLabo 32001 diffractometer	2338 reflections with *I* > 2σ(*I*)
Radiation source: fine-focus sealed tube	*R*_int_ = 0.024
graphite	θ_max_ = 25.5°, θ_min_ = 1.3°
Detector resolution: 10.0 pixels mm^-1^	*h* = −6→6
ω scans	*k* = −10→8
9663 measured reflections	*l* = −32→38
2816 independent reflections	

### Refinement

**Table:**

Refinement on *F*^2^	Primary atom site location: structure-invariant direct methods
Least-squares matrix: full	Secondary atom site location: difference Fourier map
*R*[*F*^2^ > 2σ(*F*^2^)] = 0.039	Hydrogen site location: inferred from neighbouring sites
*wR*(*F*^2^) = 0.128	H-atom parameters constrained
*S* = 1.13	*w* = 1/[σ^2^(*F*_o_^2^) + (0.0656*P*)^2^ + 0.4642*P*] where *P* = (*F*_o_^2^ + 2*F*_c_^2^)/3
2816 reflections	(Δ/σ)_max_ = 0.001
183 parameters	Δρ_max_ = 0.26 e Å^−^^3^
0 restraints	Δρ_min_ = −0.25 e Å^−^^3^

### Special details

**Table:**

Geometry. All e.s.d.'s (except the e.s.d. in the dihedral angle between two l.s. planes) are estimated using the full covariance matrix. The cell e.s.d.'s are taken into account individually in the estimation of e.s.d.'s in distances, angles and torsion angles; correlations between e.s.d.'s in cell parameters are only used when they are defined by crystal symmetry. An approximate (isotropic) treatment of cell e.s.d.'s is used for estimating e.s.d.'s involving l.s. planes.
Refinement. Refinement of *F*^2^ against ALL reflections. The weighted *R*-factor *wR* and goodness of fit *S* are based on *F*^2^, conventional *R*-factors *R* are based on *F*, with *F* set to zero for negative *F*^2^. The threshold expression of *F*^2^ > σ(*F*^2^) is used only for calculating *R*-factors(gt) *etc*. and is not relevant to the choice of reflections for refinement. *R*-factors based on *F*^2^ are statistically about twice as large as those based on *F*, and *R*- factors based on ALL data will be even larger.

### Fractional atomic coordinates and isotropic or equivalent isotropic displacement parameters (Å^2^)

**Table:**

	*x*	*y*	*z*	*U*_iso_*/*U*_eq_	
S1	0.00111 (11)	0.11114 (7)	0.06548 (2)	0.0548 (2)	
S2	−0.42287 (10)	0.19748 (8)	0.11875 (2)	0.0644 (2)	
C13	0.1963 (4)	0.6023 (2)	0.06658 (7)	0.0428 (5)	
C8	−0.0899 (3)	0.4037 (2)	0.09460 (6)	0.0416 (5)	
O1	−0.3112 (3)	0.6304 (2)	0.11057 (6)	0.0612 (5)	
C14	0.1880 (4)	0.7065 (3)	0.10221 (8)	0.0476 (5)	
C7	−0.2105 (3)	0.5153 (3)	0.12490 (7)	0.0442 (5)	
C4	−0.2015 (4)	0.4849 (3)	0.17132 (7)	0.0471 (5)	
C9	−0.1611 (4)	0.2504 (3)	0.09308 (6)	0.0433 (5)	
N1	0.1825 (4)	0.7886 (3)	0.13089 (8)	0.0688 (6)	
N2	0.4570 (5)	0.6939 (3)	0.00423 (8)	0.0775 (7)	
C12	0.0782 (4)	0.4644 (3)	0.06452 (7)	0.0436 (5)	
H12	0.1091	0.4017	0.0410	0.052*	
C15	0.3442 (4)	0.6523 (3)	0.03182 (8)	0.0531 (6)	
C3	−0.0286 (5)	0.3946 (3)	0.18964 (8)	0.0632 (7)	
H3	0.0884	0.3489	0.1730	0.076*	
C11	−0.2165 (5)	−0.0279 (3)	0.04676 (9)	0.0673 (7)	
H11A	−0.2667	−0.0931	0.0699	0.101*	
H11B	−0.1483	−0.0923	0.0249	0.101*	
H11C	−0.3502	0.0277	0.0354	0.101*	
C10	−0.3350 (6)	0.0311 (4)	0.15069 (10)	0.0833 (9)	
H10A	−0.2272	−0.0333	0.1349	0.125*	
H10B	−0.4722	−0.0293	0.1581	0.125*	
H10C	−0.2588	0.0675	0.1761	0.125*	
C5	−0.3728 (5)	0.5550 (4)	0.19670 (8)	0.0672 (7)	
H5	−0.4862	0.6200	0.1845	0.081*	
C1	−0.2049 (7)	0.4371 (5)	0.25760 (9)	0.0886 (10)	
H1	−0.2081	0.4190	0.2867	0.106*	
C6	−0.3743 (6)	0.5282 (5)	0.23948 (10)	0.0907 (10)	
H6	−0.4918	0.5726	0.2563	0.109*	
C2	−0.0300 (7)	0.3719 (4)	0.23359 (10)	0.0841 (9)	
H2	0.0881	0.3124	0.2465	0.101*	

### Atomic displacement parameters (Å^2^)

**Table:**

	*U*^11^	*U*^22^	*U*^33^	*U*^12^	*U*^13^	*U*^23^
S1	0.0588 (4)	0.0489 (4)	0.0568 (4)	−0.0146 (3)	0.0101 (3)	−0.0086 (3)
S2	0.0452 (3)	0.0742 (5)	0.0739 (5)	−0.0231 (3)	0.0133 (3)	−0.0116 (3)
C13	0.0436 (11)	0.0424 (11)	0.0423 (11)	−0.0046 (9)	−0.0006 (8)	0.0003 (9)
C8	0.0394 (10)	0.0470 (11)	0.0384 (11)	−0.0078 (9)	−0.0043 (8)	−0.0007 (9)
O1	0.0509 (9)	0.0683 (11)	0.0644 (11)	0.0137 (8)	0.0015 (8)	0.0101 (9)
C14	0.0399 (11)	0.0470 (12)	0.0560 (14)	−0.0030 (9)	−0.0046 (9)	−0.0036 (11)
C7	0.0329 (9)	0.0503 (12)	0.0493 (12)	−0.0041 (9)	−0.0017 (8)	−0.0002 (10)
C4	0.0448 (11)	0.0511 (12)	0.0453 (12)	−0.0066 (9)	−0.0003 (9)	−0.0023 (10)
C9	0.0411 (10)	0.0502 (12)	0.0384 (11)	−0.0111 (9)	−0.0021 (8)	−0.0014 (9)
N1	0.0609 (13)	0.0696 (14)	0.0758 (15)	−0.0004 (10)	−0.0015 (11)	−0.0257 (13)
N2	0.0999 (18)	0.0699 (15)	0.0628 (14)	−0.0293 (13)	0.0150 (13)	0.0073 (12)
C12	0.0479 (11)	0.0463 (12)	0.0367 (11)	−0.0061 (9)	0.0003 (8)	−0.0033 (9)
C15	0.0646 (14)	0.0431 (12)	0.0517 (14)	−0.0139 (11)	0.0008 (11)	0.0007 (11)
C3	0.0706 (16)	0.0651 (16)	0.0538 (15)	0.0063 (13)	−0.0092 (12)	−0.0026 (12)
C11	0.0893 (18)	0.0542 (14)	0.0584 (15)	−0.0278 (13)	0.0043 (13)	−0.0119 (12)
C10	0.109 (2)	0.081 (2)	0.0601 (17)	−0.0354 (18)	0.0254 (16)	0.0058 (15)
C5	0.0591 (14)	0.0854 (19)	0.0571 (16)	0.0016 (14)	0.0114 (12)	−0.0041 (14)
C1	0.116 (3)	0.107 (3)	0.0429 (15)	−0.017 (2)	0.0079 (16)	0.0082 (17)
C6	0.088 (2)	0.130 (3)	0.0549 (18)	0.003 (2)	0.0176 (16)	−0.0021 (19)
C2	0.104 (2)	0.084 (2)	0.0641 (19)	0.0032 (18)	−0.0244 (17)	0.0116 (16)

### Geometric parameters (Å, °)

**Table:**

S1—C9	1.734 (2)	C12—H12	0.93
S1—C11	1.806 (2)	C3—C2	1.397 (4)
S2—C9	1.747 (2)	C3—H3	0.93
S2—C10	1.806 (3)	C11—H11A	0.96
C13—C12	1.352 (3)	C11—H11B	0.96
C13—C14	1.431 (3)	C11—H11C	0.96
C13—C15	1.443 (3)	C10—H10A	0.96
C8—C9	1.366 (3)	C10—H10B	0.96
C8—C12	1.439 (3)	C10—H10C	0.96
C8—C7	1.510 (3)	C5—C6	1.365 (4)
O1—C7	1.220 (3)	C5—H5	0.93
C14—N1	1.142 (3)	C1—C6	1.358 (5)
C7—C4	1.485 (3)	C1—C2	1.364 (5)
C4—C3	1.371 (3)	C1—H1	0.93
C4—C5	1.391 (3)	C6—H6	0.93
N2—C15	1.135 (3)	C2—H2	0.93
			
C9—S1—C11	104.55 (12)	S1—C11—H11A	109.5
C9—S2—C10	103.13 (13)	S1—C11—H11B	109.5
C12—C13—C14	124.00 (19)	H11A—C11—H11B	109.5
C12—C13—C15	120.4 (2)	S1—C11—H11C	109.5
C14—C13—C15	115.56 (19)	H11A—C11—H11C	109.5
C9—C8—C12	121.01 (19)	H11B—C11—H11C	109.5
C9—C8—C7	119.33 (18)	S2—C10—H10A	109.5
C12—C8—C7	119.26 (18)	S2—C10—H10B	109.5
N1—C14—C13	179.3 (2)	H10A—C10—H10B	109.5
O1—C7—C4	121.3 (2)	S2—C10—H10C	109.5
O1—C7—C8	118.9 (2)	H10A—C10—H10C	109.5
C4—C7—C8	119.80 (19)	H10B—C10—H10C	109.5
C3—C4—C5	119.8 (2)	C6—C5—C4	120.0 (3)
C3—C4—C7	122.3 (2)	C6—C5—H5	120.0
C5—C4—C7	117.9 (2)	C4—C5—H5	120.0
C8—C9—S1	120.97 (16)	C6—C1—C2	120.8 (3)
C8—C9—S2	118.70 (17)	C6—C1—H1	119.6
S1—C9—S2	120.31 (12)	C2—C1—H1	119.6
C13—C12—C8	127.4 (2)	C1—C6—C5	120.3 (3)
C13—C12—H12	116.3	C1—C6—H6	119.9
C8—C12—H12	116.3	C5—C6—H6	119.9
N2—C15—C13	178.5 (3)	C1—C2—C3	119.9 (3)
C4—C3—C2	119.3 (3)	C1—C2—H2	120.1
C4—C3—H3	120.4	C3—C2—H2	120.1
C2—C3—H3	120.4		
			
C9—C8—C7—O1	−119.9 (2)	C10—S2—C9—C8	−129.17 (19)
C12—C8—C7—O1	53.0 (3)	C10—S2—C9—S1	51.99 (17)
C9—C8—C7—C4	61.1 (3)	C14—C13—C12—C8	6.1 (4)
C12—C8—C7—C4	−126.1 (2)	C15—C13—C12—C8	−174.6 (2)
O1—C7—C4—C3	−156.3 (2)	C9—C8—C12—C13	−167.0 (2)
C8—C7—C4—C3	22.7 (3)	C7—C8—C12—C13	20.3 (3)
O1—C7—C4—C5	22.0 (3)	C5—C4—C3—C2	1.2 (4)
C8—C7—C4—C5	−159.0 (2)	C7—C4—C3—C2	179.5 (2)
C12—C8—C9—S1	17.8 (3)	C3—C4—C5—C6	−2.8 (4)
C7—C8—C9—S1	−169.48 (15)	C7—C4—C5—C6	178.8 (3)
C12—C8—C9—S2	−161.03 (16)	C2—C1—C6—C5	0.5 (6)
C7—C8—C9—S2	11.7 (3)	C4—C5—C6—C1	2.0 (5)
C11—S1—C9—C8	−148.69 (19)	C6—C1—C2—C3	−2.1 (6)
C11—S1—C9—S2	30.13 (17)	C4—C3—C2—C1	1.2 (5)

### Hydrogen-bond geometry (Å, °)

**Table:**

*D*—H···*A*	*D*—H	H···*A*	*D*···*A*	*D*—H···*A*
C10—H10A···S1	0.96	2.82	3.360 (3)	116
C12—H12···S1	0.93	2.66	3.040 (2)	105
